# Supplementary material for: The Overexpression of Peanut (Arachis hypogaea L.) AhALDH2B6 in Soybean Enhances Cold Resistance
Source: Plants (Basel). 2023 Aug 12;12(16):2928. doi: 10.3390/plants12162928 (PMC10459444; doi:10.3390/plants12162928)
Supplement: Supplementary file 1 [file plants-12-02928-s001.zip › plants-2555405-supplementary.pdf]

Supplementary Materials

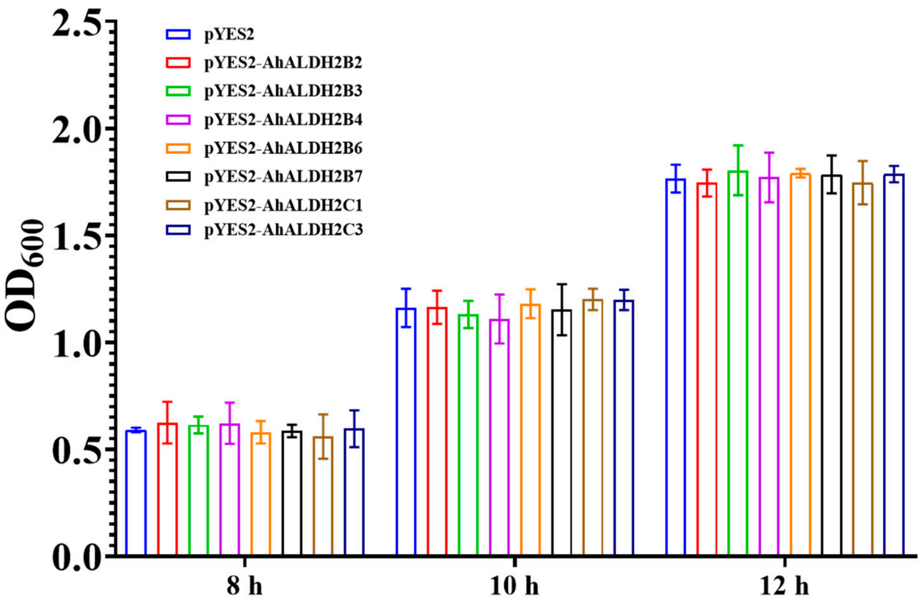

Figure S1. OD_{600} values of yeast cells at different time points under normal culture conditions.

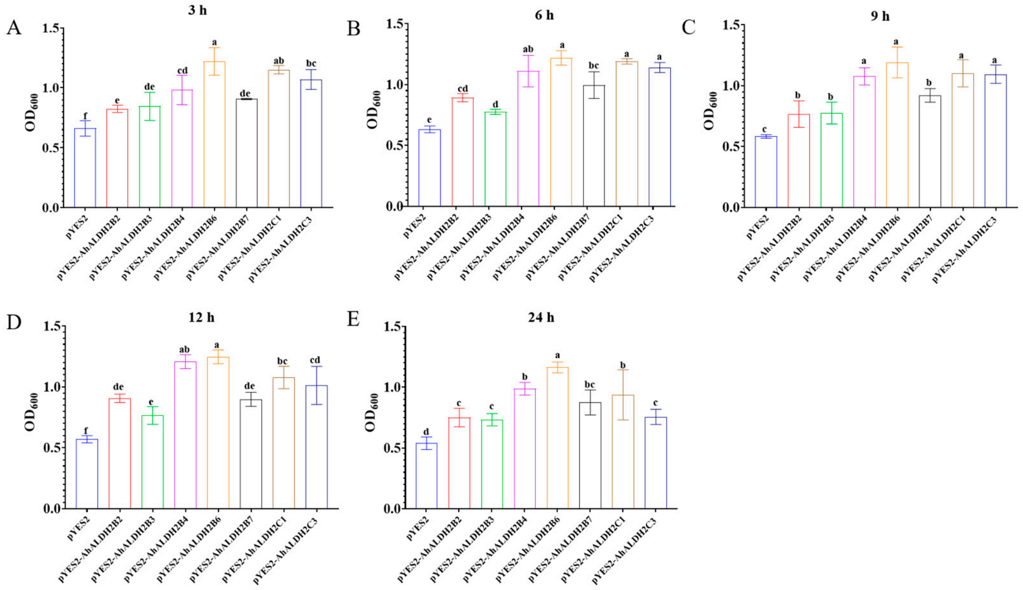

Figure S2. OD_{600} values after 8 h of resuscitation at low temperature after various treatment times. Values with different superscripts are significantly different ($P < 0.05$)

Table S1. Primers used in this study.

Primer Name	Sequence 5′to 3′
AhALDH2B6-F	ACGGGGGACTCTTGAATGACAGCCTATGAAAGGCAAAG
AhALDH2B6-R	GGGGAAATTCGAGCTCTAAAGCCAAGCAGGGTTCTTC
AhALDH2B2-F	gggaatattaagcttggtaccATGAAGATGTTTATGGGTGGCC
AhALDH2B2-R	tgatggatatctgcagaattcCCTCTCCTTTGGCATTGCTG
AhALDH2B3-F	gggaatattaagcttggtaccATGGCTGTTAGAATTCTTTCGTCTC
AhALDH2B3-R	tgatggatatctgcagaattcCTACAACCATGCTGGATTCTTCAA
AhALDH2B4-F	gggaatattaagcttggtaccATGGGTTGTTGTAAGCCTACAATATT
AhALDH2B4-R	tgatggatatctgcagaattcTCACTTATGTATTACTAAAGAATCCAT

AhALDH2B6-F2	gggaatattaagcttggtaccATGACAGCCTATGAAAGGCAAAG
AhALDH2B6-R2	tgatggatatctgcagaattcCTAAAGCCAAGCAGGGTTCTTC
AhALDH2B7-F	gggaatattaagcttggtaccATGGCTGTTAGAATTCTTTCGTCTC
AhALDH2B7-R	tgatggatatctgcagaattcCTACAACCATGCTGGATTCTTCAA
AhALDH2C3-F	gggaatattaagcttggtaccATGACAGCTCTTTCCAACGGC
AhALDH2C3-R	tgatggatatctgcagaattcTCAAAGCCAAGGAGAATTGTATATG
Bar-F	ATCGAGACAAGCACGGTCAA
Bar-R	CTGAAGTCCAGCTGCCAGAA
AhALDH2B6-F3	ATGACAGCCTATGAAAGGCAAAG
AhALDH2B6-R3	CTAAAGCCAAGCAGGGTTCTTC
Arachis-*Actin11*-F	TTGGAATGGGTCAGAAGGATGC
Arachis-*Actin11*-R	AGTGGTGCCTCAGTAAGAAGC
AhALDH2B1-qPCR-F	GTGGATTCTGCATCAGGGGAA
AhALDH2B1-qPCR-R	TAGGCCAGGGTCCTTCATCA
AhALDH2B2-qPCR-F	TGCGAGTTGTTTGAAATGCGT
AhALDH2B2-qPCR-R	ACTTTGAACGCCGGATCTGA
AhALDH2B3-qPCR-F	CCTTGCATGAGCCTATCGGT
AhALDH2B3-qPCR-R	TGGTGTTTGCTCTGCAGTTT
AhALDH2B4-qPCR-F	ATGGGGCTTTGAGTGGGTTG
AhALDH2B4-qPCR-R	CGGGAGTGTCTGTGTGTTCT
AhALDH2B5-qPCR-F	GGAATAGAGCAAGGTCCGCA
AhALDH2B5-qPCR-R	CCACGCATTTCAAACGACT
AhALDH2B6-qPCR-F	TCGAAACCGGGGGAGATAGA
AhALDH2B6-qPCR-R	GTCCATACCGCGTGTTGTTC
AhALDH2B7-qPCR-F	CCTTGCATGAGCCTATCGGT
AhALDH2B7-qPCR-R	TGGTGTCTGCTCTGCAGTTT
AhALDH2B8-qPCR-F	AGGCAGAAGGGTTACAGCAT
AhALDH2B8-qPCR-R	AGAAGAAGAAGGTGGTGCTC
AhALDH2C1-qPCR-F	ACTGCTGGTGCTGCAGTAAG
AhALDH2C1-qPCR-R	GCACATATTTCTCCCGCCTTC
AhALDH2C2-qPCR-F	TGTTGACCTTGCTCTCTTCGG
AhALDH2C2-qPCR-R	TTTTGCCTTCTCCACAACTTTTT
AhALDH2C3-qPCR-F	TGCTGATCTTGCTCTCTTGGG
AhALDH2C3-qPCR-R	TCCCTGAAATTCAAAGCCAACA
AhALDH2C4-qPCR-F	TAGTCCAGCATTGGCTGCT
AhALDH2C4-qPCR-R	TGAAGCTAACATTGAGCACACC
AhALDH2C5-qPCR-F	GGATGTCTGCCTCACAAAGGA
AhALDH2C5-qPCR-R	TGAGGGCAATGATGGACTGG
AhALDH2C6-qPCR-F	ATATGGGTTAGCAGCGGGGA
AhALDH2C6-qPCR-R	CATGTTTCCCGCGTGAGGT
AhALDH2C7-qPCR-F	GATCTTGACAAAGCCGCTGA
AhALDH2C7-qPCR-R	ACCAGCAGCACATATTTCTCCAA
AhALDH2C8-qPCR-F	TGGGCATAGCATTCTTTTCTTCC
AhALDH2C8-qPCR-R	GGTGCCATCAGCTAGAATCCC
AhALDH2C10-qPCR-F	AGACGCCATTGATGCTGGAA
AhALDH2C10-qPCR-R	TGGATCTTATCGGCAGCACC
AhALDH2C11-qPCR-F	TGAGAAGGCGAATAGCAGCA
AhALDH2C11-qPCR-R	CTGCTCGGATGGACCTTGAC
qGmActin6-F	CGGTGGTTCTATCTTGGCATC
qGmActin6-R	GTCTTTCGCTTCAATAACCCTA

Table S2. Differentially expressed up-regulated and down-regulated genes.

Up-Regulated Gene	Down-Regulated Gene
Glyma.16G170100	Glyma.01G131100
Glyma.09G185500	Glyma.13G046300
Glyma.10G189900	Glyma.06G122600
Glyma.10G246300	Glyma.19G085100
Glyma.16G210000	Glyma.19G046300
Glyma.13G347600	Glyma.03G055100
Glyma.01G119600	Glyma.13G285400
Glyma.13G237700	Glyma.04G183100
Glyma.15G128700	Glyma.07G091600
Glyma.10G193900	Glyma.10G064900
Glyma.05G207500	Glyma.03G189600
Glyma.08G014200	Glyma.13G149700
Glyma.14G141000	Glyma.19G190000
Glyma.05G208700	Glyma.01G225100
Glyma.12G088300	Glyma.15G013300
Glyma.03G189200	Glyma.07G153100
Glyma.17G008500	Glyma.13G095000
Glyma.08G362500	Glyma.12G218300
Glyma.09G010500	Glyma.06G109200
Glyma.05G198000	Glyma.16G204600
